# Biological Role of Paenilarvins, Iturin-Like Lipopeptide Secondary Metabolites Produced by the Honey Bee Pathogen *Paenibacillus larvae*

**DOI:** 10.1371/journal.pone.0164656

**Published:** 2016-10-19

**Authors:** Gillian Hertlein, Marlene Seiffert, Sebastian Gensel, Eva Garcia-Gonzalez, Julia Ebeling, Ranko Skobalj, Anja Kuthning, Roderich D. Süssmuth, Elke Genersch

**Affiliations:** 1 Institute for Bee Research, Department of Molecular Microbiology and Bee Diseases, Hohen Neuendorf, Germany; 2 Technische Universität Berlin, Institut für Chemie, Berlin, Germany; 3 Freie Universität Berlin, Fachbereich Veterinärmedizin, Institut für Mikrobiologie und Tierseuchen, Berlin, Germany; University of Milan, ITALY

## Abstract

The Gram-positive bacterium *Paenibacillus larvae* (*P*. *larvae*) is the causative agent of a deadly honey bee brood disease called American Foulbrood (AFB). AFB is a notifiable epizootic in most countries and, hence, *P*. *larvae* is of considerable relevance for veterinarians and apiculturists alike. Over the last decade, much progress has been made in the understanding of the (patho)biology of *P*. *larvae*. Recently, several non-ribosomally produced peptides (NRP) and peptide/polyketide (NRP/PK) hybrids produced by *P*. *larvae* were identified. Among these NRPs were iturin-like lipopeptides, the paenilarvins A-C. Iturins are known to exhibit strong anti-fungal activity; for some iturins, cytotoxic activity towards mammalian erythrocytes and human cancer cell lines are described. We here present our results on the analysis of the natural function of the paenilarvins during pathogenesis of *P*. *larvae* infections. We demonstrated production of paenilarvins in infected larvae. However, we could neither demonstrate cytotoxicity of paenilarvins towards cultured insect cells nor towards larvae in feeding assays. Accordingly, exposure bioassays performed with larvae infected by wild-type *P*. *larvae* and a knockout mutant of *P*. *larvae* lacking production of paenilarvins did not substantiate a role for the paenilarvins as virulence factor. Further experiments are necessary to analyze the relevance of the paenilarvins’ anti-fungal activity for *P*. *larvae* infections in the presence of fungal competitors in the larval midgut or cadaver.

## Introduction

*Paenibacillus larvae* is a Gram-positive, spore-forming bacterial entomopathogen causing a fatal brood disease of honey bees (*Apis mellifera*, *Apis cerana*) known as American Foulbrood (AFB). Early larval stages up to the age of 36 hours after egg hatching are most susceptible and become infected upon the oral uptake of *P*. *larvae* spores which are the only infectious form of this pathogen [1]. In the larval midgut, the spores germinate and the vegetative life cycle of *P*. *larvae* begins. First the bacteria lead a non-invasive, commensal-like lifestyle, colonize the larval midgut [2] and essentially consume the incoming larval food but also already start to degrade the growing peritrophic matrix by metabolizing the incorporated chitin [3–5]. This phase can last for several days. At some stage, presumably triggered by a so far unknown signal, the bacteria switch to an invasive and destructive phenotype. They attack and breach the midgut epithelium and invade the hemocoel thereby killing the larva [2]. The death of the larva does not represent the end of the vegetative lifecycle of *P*. *larvae*. Instead, the vegetative bacteria now enter a saprophytic phase during which they fully decompose the larval cadaver to a ropy mass. The bacteria in the ropy mass sporulate and the ropy mass dries up to a scale consisting of millions of spores. These spores mediate disease transmission and pathogen spread within and between colonies (for recent reviews see [6,7] and references therein). Repetitive element PCR (repPCR) performed with so-called ERIC-primers amplifying enterobacterial repetitive intergenic consensus (ERIC) sequences led to the identification of four different genotypes of *P*. *larvae*, ERIC I to IV, which were shown to differ in virulence: Depending on the ERIC genotype, infected larvae succumb to the disease within about seven (ERIC II-IV) or twelve (ERIC I) days post infection [8–10].

In the recent past, several *P*. *larvae* virulence factors have been identified, functionally characterized, and proven to be involved in AFB pathogenesis by comparing gene inactivation mutants and wild-type bacteria in infection bioassays. Among them is the chitin-degrading enzyme *Pl*CBP49 as key virulence factor [3,4], the toxins Plx1 and Plx2 specifically expressed by *P*. *larvae* ERIC I [11,12], and the ERIC II-specific S-layer protein SplA mediating *P*. *larvae* adhesion to epithelial cells [13,14]. A third toxin, originally rendered non-functional due to its genomic organization [15], was recently characterized biochemically [16] but still awaits its functional *in vivo* characterization proving that it is involved in AFB pathogenesis. Comparative whole genome sequence analysis [15] resulted in the assembly and annotation of four large gene clusters coding for the biosynthetic machineries responsible for the non-ribosomal production of peptides (NRPs) and NRP/polyketide hybrid molecules (NRP/PK) [17]. One of the identified secondary metabolites had high iron affinity and was identified as the siderophore bacillibactin [18]. Two of the identified secondary metabolites (paenilamicin and sevadicin) were novel, *P*. *larvae* ERIC II-specific molecules, which showed antibiotic activity [19,20]. A role as virulence factor could not be confirmed for any of these three secondary metabolites although paenilamicin was shown to be involved in eliminating bacterial competitors in the larval midgut [21].

One giant gene cluster, only present in the genome of *P*. *larvae* ERIC II, was demonstrated to encode the biosynthetic machinery for the paenilarvins A, B, and C, which were determined to be iturin-type lipopetides [22]. Iturins in general are reported to exhibit only limited antibacterial but strong antifungal and also hemolytic activities (for a recent review see [23]). The latter two activities have been described to rely on the ability of the iturins to interact with sterol present in eukaryotic cell membranes and to induce pore formation resulting in membrane permeabilization [24–26]. Most recently, iturin A has been described to possess moderate cytotoxic activity against different human cancer cell lines by inhibiting Akt signaling and inducing apoptosis [27,28]. As expected for members of the iturin family, paenilarvins were active against yeasts and filamentous fungi but did not show any activity against the tested Gram-positive and Gram-negative bacteria [22]. Further functional characterizations revealed that paenilarvins had cytotoxic activity against a mouse fibroblast cell line [22]. The proposed cytotoxicity of the paenilarvins led to their testing in larval toxicity assays. Feeding 5 μg purified paenilarvin A or B to four day old larvae resulted in significantly increased larval mortality for both paenilarvins compared to control larvae which were fed a standard larval diet [29] with no peptide added [22]. These results suggested that the paenilarvins have a toxic effect in honey bee larvae and, hence, might act as virulence factors for *P*. *larvae* ERIC II. No functional cytotoxins destroying the epithelial cell integrity and thus helping *P*. *larvae* ERIC II in breaching the epithelial barrier have been conclusively confirmed in *P*. *larvae* ERIC II so far [11,12,15]. Thus, paenilarvins acting as toxins during infection would close a gap in our current understanding of the pathogenic strategy of *P*. *larvae* ERIC II. Therefore, the goal of our study was to investigate this highly interesting hypothesis by analyzing the natural biological role of paenilarvins in functional *in vivo* assays.

## Results

### Identification of paenilarvin production by *P*. *larvae* in infected larvae and in cultured bacteria

**Fig 1:**
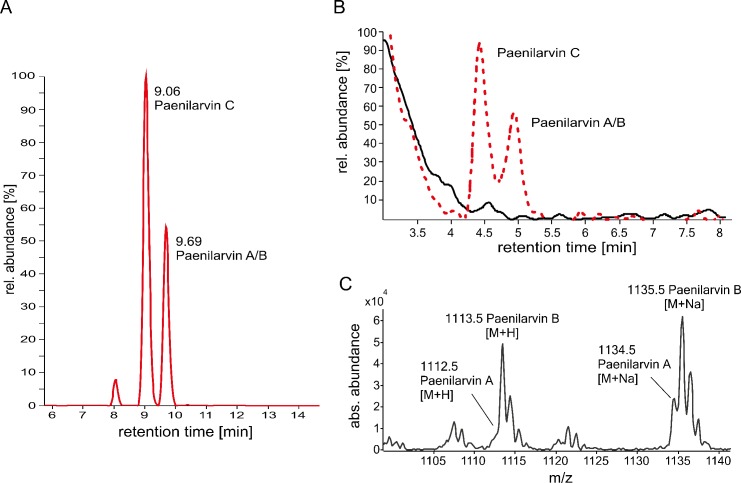
Identification of the paenilarvins in infected larvae and in *P*. *larvae* secretomes. (A) Extracted ion chromatogram (EIC) for paenilarvins detected in experimentally infected bee larvae. Paenilarvins appear at retention times R_t_ = 9.06 and R_t_ = 9.69 minutes; the peak at 8.00 min does not correspond to the paenilarvins. The figure is representative for 4 independent experiments. (B) EICs for paenilarvins A/B/C of culture supernatants of *P*. *larvae* DSM25430 wt (red dotted line) producing paenilarvins and the knockout mutant *P*. *larvae* DSM25430 Δ*itu* (solid black line) not able to produce paenilarvins. (C) Masses for paenilarvins A/B extracted from the corresponding peak (R_t_ = 5 min) in (B).

The toxicity of purified paenilarvin A and B in larval feeding assays [22] points to a putative role of paenilarvins as toxins and, hence, as virulence factors in *P*. *larvae* ERIC II infections. To experimentally address the biological role of paenilarvins during the infection process, first we confirmed the presence of paenilarvins in larvae experimentally infected with spores of the *P*. *larvae* ERIC II reference strain DSM25430 [15]. The paenilarvins were successfully detected in larval homogenates via high resolution electrospray ionization mass spectrometry (HR-ESI-MS) analyses demonstrating that they are indeed synthesized also during experimental infection (Table 1, Fig 1A). Confirmation of paenilarvin synthesis in experimentally infected larvae was a prerequisite for analyzing the role of the paenilarvins during pathogenesis via exposure bioassays performed with a gene inactivation mutant of *P*. *larvae* ERIC II, DSM25430 Δ*itu*, which did no longer produce the paenilarvins, and the corresponding wild-type strain, DSM25430 wt, expressing the paenilarvins (Fig 1B and 1C). The measured masses of the paenilarvins from infected larvae and cultured bacteria were identical (Table 1, Fig 1C).

**Table 1:** Measured and calculated nominal masses, corresponding retention times [min] and mass errors (Δppm) of the paenilarvins A, B, and C detected in infected larvae.

Retention time [min]	Compound	Measured nominal mass [M+H^+^]	Calculated nominal mass [M+H^+^]	Δppm
9.06	paenilarvin C	1084.5780	1084.5786	0.6
9.69	paenilarvin A	1112.6090	1112.6099	0.9
9.69	paenilarvin B	1113.6029	1113.5939	9.0

### Role of paenilarvins in the pathogenesis of *P*. *larvae* infections

**Fig 2:**
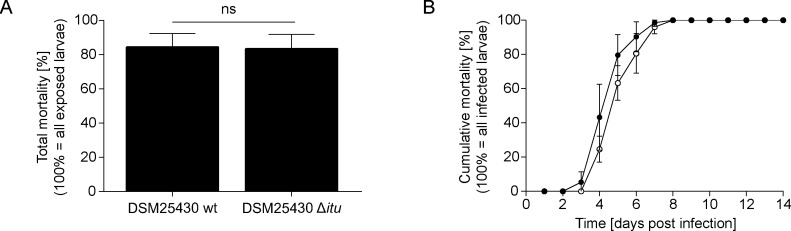
Analysis of paenilarvin as putative virulence factor during pathogenesis. (A) At the age of 12 hours after egg hatching, honey bee larvae were infected with *P*. *larvae* DSM25430 wt and DSM25430 Δ*itu*. Daily mortality due to *P*. *larvae* infection was recorded and total mortality after 15 days was determined. Bars represent the percentage of exposed larvae that died from American Foulbrood after 15 days. Bars represent mean values ± SD of three independent exposure bioassays for each group and 30 larvae per group. No significant difference in total mortality (Student´s t-test; p-value = 0.89) was observed. (B) Daily mortality due to *P*. *larvae* infection was recorded and cumulative mortality per day was calculated. Curves represent mean cumulative mortality ± SD for each day of 3 replicates with 30 larvae each. Analysis of the data by two-way ANOVA did not reveal any significant difference between the two curves (p-value = 0.15).

Wild-type and mutant bacteria were tested in parallel in exposure bioassays and total as well as cumulative mortality of infected larvae was determined. In the groups infected with wild-type *P*. *larvae*, 84.33% ± 8.08% (Fig 2A) died within eight days (Fig 2B) while the mutant bacteria killed 83.33% ± 8.51% (Fig 2A) also within eight days (Fig 2B). Hence, no significant differences in larval mortality between the groups infected with wild-type DSM25430 wt or mutant DSM25430 Δ*itu* could be detected for total (Fig 2A; Student’s t-test, p-value = 0.89) or for cumulative (Fig 2; two-way ANOVA, p-value = 0.15) mortality suggesting that paenilarvins did not act as virulence factors of *P*. *larvae* ERIC II in exposure bioassays.

**Fig 3:**
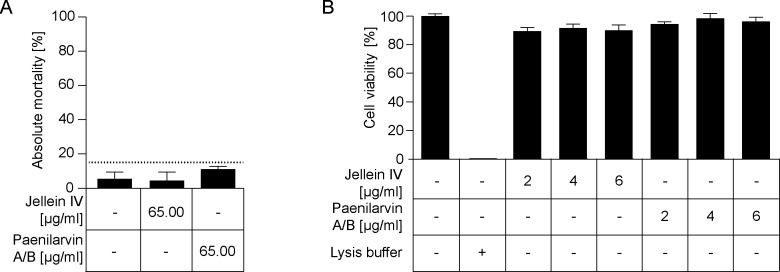
Toxicity of paenilarvins towards larvae and insect cells. (A) Larvae were *in vitro*-reared on normal larval diet. At day 4 post grafting, the treatment group received larval diet supplemented with 65 μg/mL paenilarvin A/B. Of the two negative control groups, one received larval diet supplemented 65 μg/mL jellein IV and the other received un-supplemented larval diet. Larvae were reared for 14 days and total larval mortality until day 14 is shown. The results are shown as mean values ± SD of three replicates with 30 larvae each. Larval mortality stayed below 15% for all three groups and no statistically significant difference in larval mortality between the control groups and the paenilarvin treatment group could be established (one-way ANOVA, p-value = 0.44). (B) BTI-Tn5B1-4 cells were incubated for 48 h in cell culture medium (SF900 II) without supplement or supplemented with different concentrations of jellein IV as negative control and of paenilarvin A/B isolated from DSM25430 wt. Cell lysis buffer was used as positive control. Cell viability was measured quantitatively by MTT test and the results are shown as mean values ± SEM of three replicates. No statistically significant difference in cell viability after incubation with any paenilarvin A/B concentration compared to the negative controls was evident (one-way ANOVA, p-value = 0.40).

These results were somewhat surprising considering that paenilarvin A and B had recently been shown to be toxic for honey bee larvae [22]. Therefore, we next performed larval feeding assays with purified paenilarvin A/B and exposed four day old larvae for 24 hours to 65 μg paenilarvin/mL larval food. Given that four day old larvae consume about 30 μL larval food within 24 hours [29], this concentration resulted in a dose of about 1.95 μg paenilarvin A/B taken up by each larva. In the treatment groups, 11.0% ± 1.73% larvae died until day 14 post grafting (Fig 3A). In the control groups, which received larval diet without any peptide added throughout the entire experiment, total larval mortality after 14 days was 5.33% ± 4.04% (Fig 3A). In addition, we used jellein IV as control peptide. Jelleines represent a family of four antimicrobial peptides (jelleines I–IV) present in royal jelly of honey bees. All four jelleines were shown to possess no cytolytic activity; jelleine IV also lacks antibacterial and antifungal activity exhibited by jelleine I-III [30]. In the groups which were fed with larval diet containing jellein IV at day four post grafting, 4.33% ± 5.13% of the exposed larvae died during the course of the experiment (Fig 3A). Hence, larval mortality was below 15% for all three groups (Fig 3A). Statistical analysis did not reveal a significant difference between the control groups and the paenilarvin treatment group (one-way ANOVA, p-value = 0.44) (Fig 3A). Therefore, paenilarvin A/B has no toxic effect when fed to larvae corroborating that paenilarvins might rather not act as virulence factors during *P*. *larvae* infections.

### *In vitro* cytotoxicity of paenilarvins towards cultured insect cells

Previously, the hypothesis that paenilarvins might act as virulence factors of *P*. *larvae* ERIC II was partly based on their cytotoxicity towards a mouse fibroblast cell line [22]. For this mammalian cell line, an IC_50_ of 4 μg/mL was determined [22]. Since vertebrate/mammalian and invertebrate/insect cells might differ in their susceptibility towards paenilarvins, we tested the cytotoxicity of purified paenilarvin A/B towards the insect cell line BTI-Tn5B1-4 derived from the moth *Trichoplusia ni* (Lepidoptera). This cell line has previously been used to demonstrate the cytotoxicity of the novel *P*. *larvae* secondary metabolite paenilamicin [17,20,21]. Cells were incubated for 48 hours with paenilarvin A/B or jellein IV added to the cell culture medium at different concentrations (2 μg/mL, 4 μg/mL, 6 μg/mL). While 100% of the cells incubated with cell lysis buffer (positive control) were dead after 48 hours, about 90% of the cells survived after being incubated with 2 μg/mL, 4 μg/mL, or 6 μg/mL paenilarvin A/B or jellein IV (Fig 3B). The values differed between 89.41% ± 2.56% for incubation with 2 μg/mL jellein IV and 98.33% ± 3.33% for incubation with 4 μg/mL paenilarvin A/B. No statistically significant toxic effect (one-way ANOVA, p-value = 0.40) on cell viability at any of the tested paenilarvin concentrations could be detected. This result suggested that paenilarvin A/B were not toxic for the tested insect cells. This was an experimental outcome that was consistent with the results obtained in exposure bioassays and larval feeding assays.

## Discussion

Elucidation of the molecular pathogenesis of *P*. *larvae* infections led to the identification of several virulence factors of *P*. *larvae* and the insight that the two genotypes of *P*. *larvae* distributed worldwide, ERIC I and II, developed different strategies to attack and kill larvae (for a recent review see [7]). The first steps of the infection process are well described for both genotypes. However, only for *P*. *larvae* ERIC I virulence factors attacking the epithelial cells have been identified. For *P*. *larvae* ERIC II, the steps following bacterial adhesion to the epithelial cell layer and the proteins involved in attacking host cells still remain elusive. In a recent publication [22] the hypothesis was put forward that paenilarvins, iturin-type lipopeptides produced by the bee pathogen *P*. *larvae*, act as cytotoxins and represent *P*. *larvae* ERIC II-specific virulence factors. No functional toxins have been found in the genome of *P*. *larvae* ERIC II so far and, therefore, the hypothesis that paenilarvins might fill in this position was tempting. To directly validate the role of paenilarvins during *P*. *larvae* infection of honey bee larvae, we generated a *P*. *larvae* ERIC II gene inactivation mutant lacking the production of all paenilarvin derivatives (Fig 1). Surprisingly, the absence of paenilarvin production did not affect the ability of *P*. *larvae* ERIC II to kill larvae. Neither total mortality of larvae (Fig 2A) nor disease progression in larvae (Fig 2B) was significantly altered when larvae were infected with the paenilarvin deficient strain compared to the lethality of the wild-type bacteria. These results contrasted our initial hypothesis and did not fit to the results obtained in the paenilarvin feeding assay presented recently [22]. Based on the famous citation of Paracelsus: “Sola dosis facit venenum” (Only the dose makes the poison) we considered that a dose of 5 μg paenilarvins per larva as used by Sood and co-workers [22] might have been artificially high. And indeed, according to our estimation based on the number of *P*. *larvae* present in larvae at different time points post infection and the amount of paenilarvins produced per bacterium, the amount of paenilarvin produced during infection may vary between 0.75 ng and 46.5 ng. Hence, a dose of 5 μg paenilarvin per larva is >100 fold of the maximum dose estimated to occur naturally. Even a dose of 1.95 μg paenilarvin A/B taken up per larva in our feeding assays exceeded the estimated maximal paenilarvin concentration per larva by around 40 fold. However, even with this rather high paenilarvin dose per larva, larval mortality was below 15% in the feeding assays which corresponds to the “natural mortality” of larvae in the hive [29,31]. We did not test higher dosages because a closer look at the data published by Sood and co-workers [22] revealed that the three replicates of the published feeding assays differed considerably and that in some replicates of the control groups, mortality was even higher than in the treatment groups. In addition, in the published assays mortality in the control groups differed between 30% and close to 50% and, therefore, mortality in all control replicates was above the threshold of 20% that is considered tolerable for artificial rearing of larvae. It is consensus that all experiments involving artificial rearing of larvae which have a control mortality exceeding 20% should be invalidated [29]. In our feeding assays, mortality in the control groups as well as in the treatment group was <15% after 14 days of artificial rearing of larvae. Likewise, total mortality in the control groups of the laboratory infection experiments was 8.8% ± 1.7% at day 15. Therefore, all these experiments are valid and we have to conclude that we could not confirm that paenilarvins act as cytotoxins during infection.

Iturins have been described to mediate toxicity towards fungal cells by interacting with cell membrane components leading to pore formation and cell permeabilization [24,25,32]. In addition, some iturin-type lipopeptides have been described to have hemolytic activity and to permeabilize erythrocyte cell membranes [26,33]. However, *P*. *larvae* ERIC II grown on Columbia sheep blood agar (CSA) plates does not show hemolytic activity [9] suggesting that paenilarvins do not act as cytotoxins for erythrocytes or that paenilarvins are not produced when cultured on agar plates. Still, to further substantiate the negative outcome of our exposure bioassays and larval feeding assays, we also performed cell toxicity assays with an insect cell line already successfully used to demonstrate the cytotoxicity of paenilamicin [20], another secondary metabolite of *P*. *larvae* [17,21]. Because iturins interact with cell membrane components which might differ between mammalian and insect cells, a difference in susceptibility between these cell types towards a certain lipopeptide seemed likely. Indeed, in contrast to the mouse fibroblast cell line [22], the insect cells tested were unaffected by 48 hours of incubation with paenilarvins even at a concentration of 6 μg/mL which exceeded the IC_50_ of 4 μg/mL determined for the mouse cells. Although this result again contradicted previously published data [22] they match the data from exposure bioassays and larval feeding assays presented in the study at hand and contribute to a consistent picture: Despite their cytotoxic activity on mammalian cells, the paenilarvins produced by *P*. *larvae* were not toxic towards the tested insect cells and were not involved as virulence factors in pathogenesis of *P*. *larvae* infections.

How does this revised view on the biological role of paenilarvins fit into the present literature on iturin-type lipopeptide secondary metabolites? In general, iturins are produced by members of the genera *Bacillus*, *Paenibacillus*, *Burkholderia*, *Serratia* and *Pseudomonas*. Some of the producing species are known (entomo)pathogens. However, to the best of our knowledge, no iturin-like lipopeptide has ever been identified as essential factor for the virulence of the producing bacterium. Iturins are best known for their strong antifungal activity [34], a feature which was also proven for the paenilarvins [22]. Therefore, it is conceivable that paenilarvins are involved in eliminating fungal competitors in the midgut of the larva still living or during the saprophytic phase of life of *P*. *larvae*. For the anti-bacterially active NRP/PK hybrid paenilamicin, produced by *P*. *larvae* during infection, it has already been shown that it antagonizes the saprophytic bacterium *Paenibacillus alvei*, thus helping *P*. *larvae* to establish a pure culture in the diseased larva [20,21]. The antifungal activity of paenilarvins might also include activity against the nearly ubiquitously occurring bee pathogenic fungus *Ascosphaera apis* (etiological agent of chalkbrood) [35]. Further experiments are necessary to address the interesting question of the relevance of paenilarvins’ anti-fungal activity during pathogenesis of *P*. *larvae* infections.

## Material and Methods

### Bacterial strain and culture conditions

The broadly characterized and recently sequenced type strain of *Paenibacillus larvae* genotype ERIC II, strain DSM25430, was used in this study [9,15]. Bacteria were cultivated either in Mueller-Hinton-yeast-phosphate-glucose-pyruvate (MYPGP) liquid broth [36] or on CSA plates (Oxoid, Hampshire, UK) at 37°C. Preparation of *P*. *larvae* spore suspensions and determination of spore concentration via cultivating serial dilutions on CSA were carried out as previously described [8].

### Detection of paenilarvins in infected larvae via HR-ESI-MS

To confirm the presence of paenilarvins in experimentally infected larvae, laboratory infection assays (exposure bioassays) were performed essentially as described [8,9,29]. Briefly, first instar honey bee larvae were grafted into wells of 24-well-plates already containing normal (control groups) artificial larval diet (66% royal jelly (v/v), 33% glucose (w/v) and 33% fructose (w/v)) or larval diet contaminated with spores of DSM25430 at a concentration of 500 colony forming units (cfu)/mL larval diet, which was determined in prior experiments to correspond to the LC_80_ (lethal concentration) of this strain. Larvae in the infection groups were exposed to infectious diet during the first 24 hours after grafting and obtained normal larval diet thereafter. Larvae in the control groups were fed with normal larval diet throughout the entire experiment. Representatives of the genotype *P*. *larvae* ERIC II kill the first larvae already at day 3 post infection [8,37]. Therefore, AFB-dead larvae were collected days 3 and 4 post infection (2 individuals each), immediately frozen and stored at -20°C until analysis. For high resolution electrospray ionization mass spectrometry (HR-ESI-MS) analyses, larvae were thawed and homogenized in 200 μL of 100% acetonitrile with a pipette tip and by sonication (10 min). Homogenized bee larvae were extracted three times with 100% acetonitrile by 10 min sonication, following centrifugation for 15 min at 14.000 rpm. The supernatants were pooled and dried *in vacuo*. For mass spectrometry (MS) analytics, dried extracts were resolved in 1 mL 50% aqueous acetonitrile with 0.1% formic acid. 5 μL were subjected to HR-ESI-MS analytics performed on an Exactive Orbitrap mass spectrometer (Thermo Scientific, Bremen, Germany) in medium resolution mode, coupled to an Agilent 1200 HPLC system (Agilent Technologies. Waldbronn, Germany). Samples were analyzed by linear gradient elution using H_2_O + 0.1% formic acid as solvent A and acetonitrile + 0.1% formic acid as solvent B. The gradient was from 30% to 100% solvent B in 16 min with a 4 min isocratic elution at 100% for solvent B. The column was equilibrated with 30% B for 2 min.

### Construction of a *P*. *larvae* paenilarvin gene cluster inactivation mutant

**Fig 4:**
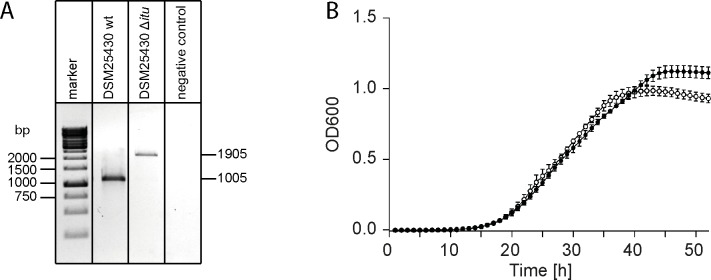
Verification of the paenilarvin gene cluster inactivation mutant *P*. *larvae* DSM25430 Δ*itu*. (A) PCR analysis of the genomic region of *P*. *larvae* DSM25430 Δ*itu* with primers flanking the insertion site in the malonyl CoA-ACP transacylase gene of the paenilarvin gene cluster revealed successful intron insertion. The amplicons of the wild-type strain and of the mutant strain carrying the insertion migrated at their expected sizes of 1005 bp and 1905 bp, respectively. (B) Growth curves of the wild-type strain *P*. *larvae* DSM25430 wt (closed circles) and the mutant strain DSM25430 *Δitu* (open circles) in liquid broth did not differ significantly (two-way-ANOVA, p-value = 0.15). Data points represent the mean ± SEM and consisted of three biological replicates with three technical replicates each.

The generation of the paenilarvin gene cluster inactivation mutant DSM25430 Δ*itu* was achieved via a recently described strategy [14,38] which is based on the TargeTron Gene Knockout System (Sigma-Aldrich, Germany). Targeted group II intron insertion at position 191 from the start codon of the malonyl CoA-acyl carrier protein (ACP) transacylase gene (ERIC2_c18780; NCBI acc. no. WP_024094097.1; [15]) of the paenilarvin gene cluster was achieved by transforming the targetron vector pTT_*itu* into *P*. *larvae*. For constructing the vector pTT_*itu*, the LI.LtrB targetron of the vector pTT_*wsf*A243 [38] was modified by primers which had been predicted by a computer algorithm (http://www.sigma-genosys.com/targetron) to ensure high insertion efficiency of the 900 bp intron at the target site in the malonyl CoA-ACP transacylase gene of the paenilarvin gene cluster. Retargeting of the vector, generation of electrocompetent *P*. *larvae* cells, and transformation of *P*. *larvae* DSM25430 wild-type bacteria to obtain the *P*. *larvae* DSM25430 Δ*itu* mutant strain was performed essentially as already described for several *P*. *larvae* genes [4,14,18,39]. The insertion was verified by PCR analysis of the corresponding genomic region using primers flanking the intron insertion site (Fig 4A). Primers used for the generation of the knockout mutant and for PCR verification of intron insertion are given in Table 2. The PCR for knockout generation was carried out according to the manufacturer’s instructions (TargeTron Gene Knockout System, Sigma-Aldrich, Germany). PCR verification of intron insertion was performed with a reaction mix containing 2 μl of genomic DNA, 200 μM peqGOLD dNTP mix (peqlab, Erlangen, Germany), 0.4 μM of primers each, and 0.5 U of HotStart Taq Plus polymerase (QIAGEN, Hilden, Germany) in a volume of 25 μl by using a thermal cycler with the following parameters: 95°C for 5 min; 40 cycles at 94°C for 1 min, 58°C for 1 min, and 72°C for 1 min; and a final extension at 72°C for 10 min. Analysis of the growth characteristics of the mutant strain in comparison to the corresponding wild-type strain in liquid broth did not reveal any significant differences between the two strains (three biological replicates with three technical replicates each; two-way ANOVA; p-value = 0.15) (Fig 4B). Wild-type and mutant DSM25430 were cultivated in liquid broth and the culture supernatant containing the bacterial secretome was collected as previously described [20]. The secretomes of the two strains were analyzed by an Agilent 6460 Triple Quadropole LC/MS system coupled to an UHPLC (Ultra High Pressure Liquid Chromatography) 1290 Infinity-Series (Agilent Technologies, Waldbronn, Germany). To this end, extracts were dissolved in 50% aqueous acetonitrile and a 5 μL portion was injected to the UHPLC column. For separation, a GRACE Vision HT C_18_ column (50x2 mm, particle size 1.5 μm) was used with linear gradient elution using H_2_O + 0.1% formic acid as solvent A and acetonitril + 0.1% formic acid as solvent B. The gradient was from 5% to 100% solvent B in 10 min, ending at 100% B isocratic for 2 min at a flow rate of 0.3 mL/min. Absence of masses in the Extracted Ion Chromatogram (EIC) corresponding to the paenilarvins confirmed the successful prevention of paenilarvin production in the knockout strain (Fig 1B).

**Table 2:** Primers used for generating and verifying the paenilarvin gene cluster inactivation mutant.

Primer name	Primer sequence	Used for
IBS_*itu*_683	5’-AAAAAAGCTTATAATTATCCTTATGCCCCGCCGGCGTGCGCCCAGATAGGGTG-3’	Paenilarvin knockout construction
EBS1d_*itu*_683	5’-CAGATTGTACAAATGTGGTGATAACAGATAAGTCGCCGGCTATAACTTACCTTTCTTTGT-3’	
EBS2_*itu*_683	5’-TGAACGCAAGTTTCTAATTTCGGTTGGGCATCGATAGAGGAAAGTGTCT-3’	
Itu290_FW	5’-TTTGTTTTGGCCAAGAGGCG-3’	Screening for intron inserting
Itu290_RV	5’-CTCTTGGGCCAGTTGCAGTT-3’	

### Exposure bioassays

To analyze the role of the paenilarvins during infection, laboratory infection assays (exposure bioassays) were performed essentially as described [8,9,29]. Briefly, first instar honey bee larvae were grafted into wells of 24-well-plates already containing normal (control groups) artificial larval diet (see above) or larval diet contaminated with spores of DSM25430 or DSM25430Δ*itu* (infection groups). In order to be able to monitor a decrease as well as an increase in larval mortality, the larvae were exposed to a spore concentration of 500 cfu/mL larval diet, which was determined in prior experiments to correspond to the LC_80_ (lethal concentration) of the wild-type strain. Larvae in the infection groups were exposed to infectious diet during the first 24 hours after grafting and obtained normal larval diet thereafter. Larvae in the control groups were fed with normal larval diet throughout the entire experiment. Larval health status and mortality were monitored daily for 14 days. During larval development, living larvae were transferred to new wells containing larval diet every day. With the beginning of the pupal development, larvae that were still alive were transferred into wells not containing any food but lined with filter paper. Dead larvae or pupae were immediately removed, streaked out on CSA, and incubated for two days at 37°C. Larvae were only classified as having died from AFB when vegetative *P*. *larvae* could be cultivated from the larval remains. Calculations of total and cumulative mortality are based on these AFB-dead larvae only. Three independent exposure bioassays were performed, each with three groups (one control group, two infection groups) and 30 larvae per group. Total mortality and cumulative mortality were analyzed with Student´s t-test and two-way ANOVA, respectively. Mortality in the control groups was 8.8% ± 1.7%, hence, even below the threshold of 15% considered as natural larval mortality and used as “quality indicator” for the validity of the experiments [29,31].

### Isolation and purification of paenilarvins A/B

*P*. *larvae* DSM25430 was fermented on a 2 L scale (as described before) and the culture supernatant was treated with 10% w/V XAD-16 amberlite adsorber resin (Sigma, St. Louis, MO, USA) and stirred at 4°C for 12 hours. The resin was filtered off and washed with 200 mL of H_2_O before the resin was stirred in 1.8 L of MeOH for 12 hours at 4°C. After filtration the MeOH was evaporated under reduced pressure and to the residue were added 25 mL of H_2_O. After freeze-drying, 3.7 g of crude material were obtained. To this material were added 30 mL of H_2_O and the aqueous phase was extracted three times with *n*BuOH (30 mL each) to obtain 760 mg of a yellowish solid after solvent-evaporation. Further purification was carried out using an Agilent 1200 Series HPLC-System (Agilent Technologies, Waldbronn, Germany) with a semi-preparative C_18_ column (50 x 2 mm, particle size 3 μm). The separation was accomplished by linear gradient elution using H_2_O + 0.1% formic acid as solvent A and acetonitril + 0.1% formic acid as solvent B. The gradient was from 30% to 70% solvent B in 12.5 min, isocratically ending at 100% B and a hold for 2 min (flow rate: 20 mL/min). Paenilarvin C (t_R_ = 7.6 min) was separated from paenilarvins A/B (t_R_ = 9.7 min) under these conditions and the paenilarvin A to B ratio was found to be 2:1, determined by integration of the corresponding UV-signals from the HPLC UV-detector. Paenilarvin A/B containing fractions were pooled and freeze-dried to give 7 mg of a white solid. Instead of further separating paenilarvin A and B, this naturally occurring paenilarvin A/B mixture was used for further experiments.

### Larval feeding assays

To analyze the toxicity of paenilarvins for honey bee larvae, larval feeding assays with purified paenilarvin A/B were performed. Firstly, we calculated the minimally and maximally possible paenilarvin A/B concentration per larva. 1.5 mg paenilarvin A/B could reproducibly be isolated from 700 mL supernatant of a bacterial culture containing 1.9E+11 *P*. *larvae* cells. Hence, one *P*. *larvae* cell roughly contributed 7.5E-6 ng paenilarvin A/B to the total yield. To obtain the number of bacterial cells per larva, bacteria were counted in ten dead larvae at days 4, 5, 6, and 7 post infection (p. i.). The least bacteria were present at day 4 p. i. (cfu_min_ = 1.0E+5; cfu_max_ = 1.3E+6) and the maximum of bacteria was counted in larvae at day 7 p. i. (cfu_min_ = 4.5E+6; cfu_max_ = 6.2E+6) corresponding to a minimum concentration of paenilarvin of 0.75 ng per larva and a maximum concentration of paenilarvin of 46.5 ng per larva.

For performing the feeding assays, first instar honey bee larvae were grafted into wells of 24-well-plates already containing normal artificial larval diet. Honey bee larvae were reared for 14 days exactly as described above for the exposure bioassays. At day 4 post grafting, larvae were fed normal larval diet which was not supplemented at all (negative control group) or which was supplemented with 65.0 μg paenilarvin A/B purified from *P*. *larvae* culture supernatants per mL larval diet (treatment group) or with 65.0 μg of the royal jelly inherent peptide jellein IV purchased from peptides&elephants (Potsdam, Germany) per mL larval diet (peptide control group). Larvae were allowed to consume this supplemented diet for 24 hours; thereafter, normal larval diet was fed until the beginning of pupal development. Larvae were examined every day; dead larvae were recorded and removed. Four day old larvae have been described to consume 30 μL larval food in 24 hours [29]. We, therefore, estimate that each larva consumed 1.95 μg paenilarvin A/B or jellein IV. This paenilarvin concentration was 2,600 times higher than the minimum and 40 times higher than the maximum calculated concentration of paenilarvin (0.75 ng per larva at day 4 p. i. and 46.5 ng per larva at day 7 p. i., see above) to make sure to not overlook any toxic effects caused by an insufficient dose. For each experimental group, three independent experiments with 30 larvae each were performed. Statistical analysis of the results was performed with one-way ANOVA.

### Cell cytotoxicity assay

The putative *in vitro* cytotoxicity of paenilarvin was tested by using the insect cell line BTI-Tn5B1-4 (derived from *Trichoplusia ni*, Lepidoptera) obtained from the *Deutsche Sammlung von Mikroorganismen und Zellkulturen* (DSMZ, Braunschweig, Germany). Cell maintenance and cytotoxicity assays were performed essentially as described before [20]. Paenilarvin A/B purified from culture supernatants (see above) was dissolved in water at a concentration of 5 mg/mL and stored at -80°C until further use. The royal jelly inherent antimicrobial peptide jellein IV [30] was used as a negative control peptide. For cytotoxicity assays, BTI-Tn5B1-4 cells were incubated for 48 h either in SF 900 II medium (Lonza, Walkersville, MD, USA) or in SF 900 II medium supplemented with purified paenilarvin A/B (treatment) or jellein IV (negative peptide control). Supplementing the medium with cell lysis buffer (99.4 ml DMSO, 0.6 mL acetic acid, 10% SDS w/v) served as positive control. The peptides were added to the medium at the following final concentrations: 2 μg/mL, 4 μg/mL, and 6 μg/mL. Cell viability was checked by MTT assay performed essentially as already described [20]. This assay is based on the ability of living cells to reduce the water soluble, yellow tetrazolium dye 3-(4,5-dimethylthiazol-2-yl)-2,5-diphenyltetrazolium bromide (MTT) to the purple colored insoluble formazan. The data shown are based on three biological replicates with three technical replicates each. Data were statistically analyzed using one-way ANOVA.

## References

[1] Tarr HLA (1937) Studies on American foulbrood of bees. I. The relative pathogenicity of vegetative cells and endospores of *Bacillus larvae* for the brood of the bee. Ann Appl Biol 24: 377–384.

[2] Yue D, Nordhoff M, Wieler LH, Genersch E (2008) Fluorescence *in situ*-hybridization (FISH) analysis of the interactions between honeybee larvae and *Paenibacillus larvae*, the causative agent of American foulbrood of honeybees (*Apis mellifera*). Environ Microbiol 10: 1612–1620. 10.1111/j.1462-2920.2008.01579.x 18331334

[3] Garcia-Gonzalez E, Genersch E (2013) Honey bee larval peritrophic matrix degradation during infection with *Paenibacillus larvae*, the aetiological agent of American foulbrood of honey bees, is a key step in pathogenesis. Environ Microbiol 15: 2894–2901. 10.1111/1462-2920.12167 23809335

[4] Garcia-Gonzalez E, Poppinga L, Fünfhaus A, Hertlein G, Hedtke K, Jakubowska A, et al (2014) *Paenibacillus larvae* chitin-degrading protein *Pl*CBP49 is a key virulence factor in American Foulbrood of honey bees. PLoS Path 10: e1004284. 10.1371/journal.ppat.1004284 PMC4117609 25080221

[5] Neuendorf S, Hedtke K, Tangen G, Genersch E (2004) Biochemical characterization of different genotypes of *Paenibacillus larvae* subsp. *larvae*, a honey bee bacterial pathogen. Microbiology 150: 2381–2390. 10.1099/mic.0.27125-0 15256579

[6] Genersch E (2010) American Foulbrood in honeybees and its causative agent, *Paenibacillus larvae*. J Invertebr Pathol 103: S10–S19. 10.1016/j.jip.2009.06.015 19909971

[7] Poppinga L, Genersch E (2015) Molecular pathogenesis of American Foulbrood: how *Paenibacillus larvae* kills honey bee larvae. Curr Opin Insect Sci 10: 29–36. 29588011 10.1016/j.cois.2015.04.013

[8] Genersch E, Ashiralieva A, Fries I (2005) Strain- and genotype-specific differences in virulence of *Paenibacillus larvae* subsp. *larvae*, the causative agent of American foulbrood disease in honey bees. Appl Environ Microbiol 71: 7551–7555. 10.1128/AEM.71.11.7551-7555.2005 16269801 PMC1287710

[9] Genersch E, Forsgren E, Pentikäinen J, Ashiralieva A, Rauch S, Kilwinski J, et al (2006) Reclassification of *Paenibacillus larvae* subsp. *pulvifaciens* and *Paenibacillus larvae* subsp. *larvae* as *Paenibacillus larvae* without subspecies differentiation. Int J Syst Evol Microbiol 56: 501–511. 10.1099/ijs.0.63928-0 16514018

[10] Rauch S, Ashiralieva A, Hedtke K, Genersch E (2009) Negative correlation between individual-insect-level virulence and colony-level virulence of *Paenibacillus larvae*, the etiological agent of American foulbrood of honeybees. Appl Environ Microbiol 75: 3344–3347. 10.1128/AEM.02839-08 19304833 PMC2681656

[11] Fünfhaus A, Ashiralieva A, Borriss R, Genersch E (2009) Use of suppression subtractive hybridization to identify genetic differences between differentially virulent genotypes of *Paenibacillus larvae*, the etiological agent of American Foulbrood of honeybees. Environ Microbiol Rep 1: 240–250. 10.1111/j.1758-2229.2009.00039.x 23765853

[12] Fünfhaus A, Poppinga L, Genersch E (2013) Identification and characterization of two novel toxins expressed by the lethal honey bee pathogen *Paenibacillus larvae*, the causative agent of American foulbrood. Environ Microbiol 15: 2951–2965. 10.1111/1462-2920.12229 23992535

[13] Fünfhaus A, Genersch E (2012) Proteome analysis of *Paenibacillus larvae* reveals the existence of a putative S-layer protein. Environ Microbiol Rep 4: 194–202. 10.1111/j.1758-2229.2011.00320.x 23757273

[14] Poppinga L, Janesch B, Fünfhaus A, Sekot G, Garcia-Gonzalez E, Hertlein G, et al (2012) Identification and functional analysis of the S-layer protein SplA of *Paenibacillus larvae*, the causative agent of American Foulbrood of honey bees. PLoS Path 8: e1002716. 10.1371/journal.ppat.1002716 PMC3355101 22615573

[15] Djukic M, Brzuszkiewicz E, Fünfhaus A, Voss J, Gollnow K, Poppinga L, et al (2014) How to kill the honey bee larva: Genomic potential and virulence mechanisms of *Paenibacillus larvae*. PLoS ONE 9: e90914 10.1371/journal.pone.0090914 24599066 PMC3944939

[16] Krska D, Ravulapalli R, Fieldhouse RJ, Lugo MR, Merrill AR (2015) C3larvin toxin, an ADP-ribosyltransferase from *Paenibacillus larvae*. J Biol Chem 290: 1639–1653. 10.1074/jbc.M114.589846 25477523 PMC4340408

[17] Müller S, Garcia-Gonzalez E, Genersch E, Süssmuth RD (2015) Involvement of secondary metabolites in the pathogenesis of the American foulbrood of honey bees caused by *Paenibacillus larvae*. Nat Prod Rep 32: 765–778. 10.1039/c4np00158c 25904391

[18] Hertlein G, Müller S, Garcia-Gonzalez E, Poppinga L, Süssmuth RD, et al (2014) Production of the catechol type siderophore bacillibactin by the honey bee pathogen *Paenibacillus larvae*. PLoS ONE 9: e108272 10.1371/journal.pone.0108272 25237888 PMC4169593

[19] Garcia-Gonzalez E, Müller S, Ensle P, Süssmuth RD, Genersch E (2014) Elucidation of sevadicin, a novel nonribosomal peptide secondary metabolite produced by the honey bee pathogenic bacterium *Paenibacillus larvae*. Environ Microbiol 16: 1297–1309. 25118351

[20] Garcia-Gonzalez E, Müller S, Hertlein G, Heid NC, Süssmuth RD, et al (2014) Biological effects of paenilamicin, a secondary metabolite antibiotic produced by the honey bee pathogenic bacterium *Paenibacillus larvae*. MicrobiologyOpen 3: 642–656. 10.1002/mbo3.195 25044543 PMC4234257

[21] Müller S, Garcia-Gonzalez E, Mainz A, Hertlein G, Heid NC, Mösker E, et al (2014) Paenilamicin—structure and biosynthesis of a hybrid non-ribosomal peptide/ polyketide antibiotic from the bee pathogen *Paenibacillus larvae*. Angew Chem Int Ed Engl 53: 10547–10828. 10.1002/anie.201404572 25080172

[22] Sood S, Steinmetz H, Beims B, Mohr KI, Stadler M, Djukic M, et al (2014) Paenilarvins: Iturin family lipopeptides from the honey bee pathogen *Paenibacillus larvae*. Chem BioChem 15: 1947–1955. 10.1002/cbic.201402139 25069424

[23] Cochrane SA, Vederas JC (2016) Lipopeptides from *Bacillus* and *Paenibacillus* spp.: A gold mine of antibiotic candidates. Med Res Rev 36: 4–31. 10.1002/med.21321 24866700

[24] Maget-Dana R, Peypoux F (1994) Iturins, a special class of pore-forming lipopeptides: biological and physicochemical properties. Toxicology 87: 151–174. 8160184 10.1016/0300-483x(94)90159-7

[25] Thimon L, Peypoux F, Walllach J, Michel G (1995) Effect of the lipopeptide antibiotic, iturin A, on morphology and membrane ultrastructure of yeast cells. FEMS Microbiol Lett 128: 101–106. 7750727 10.1111/j.1574-6968.1995.tb07507.x

[26] Aranda FJ, Teruel JA, Ortiz A (2005) Further aspects on the hemolytic activity of the antibiotic lipopeptide iturin A. Biochim Biophys Acta 1713: 51–56. 10.1016/j.bbamem.2005.05.003 15949788

[27] Dey G, Bharti R, Dhanarajan G, Das S, Dey KK, Kumar BNP, et al (2015) Marine lipopeptide Iturin A inhibits Akt mediated GSK3β and FoxO3a signaling and triggers apoptosis in breast cancer. Sci Rep 5: 10316 10.1038/srep10316 25974307 PMC4431395

[28] Son S, Ko S-K, Jang M, Kim JW, Kim GS, Lee JK, et al (2016) New cyclic lipopeptides of the iturin class produced by saltern-derived *Bacillus* sp. KCB14S006. Mar Drugs 14: 72. 10.3390/md14040072 PMC4849076 27049393

[29] Crailsheim K, Brodschneider R, Aupinel P, Behrens D, Genersch E, Vollmann J, et al (2013) Standard methods for artificial rearing of *Apis mellifera* larvae. J Apicult Res 52: 1–15.

[30] Fontana R, Mendes MA, de Souza BM, Konno K, Cesar LM, Malaspina O, et al (2004) Jelleines: a family of antimicrobial peptides from the royal jelly of honeybees (*Apis mellifera*). Peptides 25: 919–928. 10.1016/j.peptides.2004.03.016 15203237

[31] Fukuda H, Sakagami SF (1968) Worker brood survival in honey bees. Res Popul Ecol 10: 31–39.

[32] Maget-Dana R, Ptak M, Peypoux F, Michel G (1985) Pore-forming properties of iturin A, a lipopeptide antibiotic. Biochim Biophys Acta 815: 405–409. 3995034 10.1016/0005-2736(85)90367-0

[33] Quentin MJ, Besson F, Peypoux F, Michel G (1982) Action of peptidolipidic antibiotics of iturin group on erythrocytes. Effects of some lipids on hemolysis. Biochim Biophys Acta 684: 207–211. 7055563 10.1016/0005-2736(82)90007-4

[34] Besson F, Peypoux F, Quentin MJ, Michel G (1984) Action of antifungal peptidolipids from Bacillus subtilis on the cell membrane of Saccharomyces cerevisiae. J Antibiotics 37: 172–177. 6423598 10.7164/antibiotics.37.172

[35] Jensen AB, Aronstein K, Flores JM, Vojvodic S, Palacio MA, et al (2013) Standard methods for fungal brood disease research. J Apicult Res 52. 10.3896/IBRA.1.52.1.13 PMC3816652 24198438

[36] Dingman DW, Stahly DP (1983) Medium promoting sporulation of *Bacillus larvae* and metabolism of medium components. Appl Environ Microbiol 46: 860–869. 16346399 10.1128/aem.46.4.860-869.1983 PMC239480

[37] Ashiralieva A, Genersch E (2006) Reclassification, genotypes, and virulence of *Paenibacillus larvae*, the etiological agent of American foulbrood in honeybees—a review. Apidologie 37: 411–420.

[38] Zarschler K, Janesch B, Zayni S, Schäffer C, Messner P (2009) Construction of a gene knockout system for application in *Paenibacillus alvei* CCM 2051T, exemplified by the S-layer glycan biosynthesis initiation enzyme WsfP. Appl Environ Microbiol 75: 3077–3085. 10.1128/AEM.00087-09 19304819 PMC2681630

[39] Poppinga L, Genersch E (2012) Heterologous expression of green fluorescent protein in *Paenibacillus larvae*, the causative agent of American Foulbrood of honey bees. J Appl Microbiol 112: 430–435. 10.1111/j.1365-2672.2011.05214.x 22151200

